# Anti-inflammatory potential of *Capparis spinosa* L*.* in vivo in mice through inhibition of cell infiltration and cytokine gene expression

**DOI:** 10.1186/s12906-017-1569-7

**Published:** 2017-01-31

**Authors:** Khadija El Azhary, Nadia Tahiri Jouti, Meryam El Khachibi, Mouna Moutia, Imane Tabyaoui, Abdelhalim El Hou, Hafid Achtak, Sellama Nadifi, Norddine Habti, Abdallah Badou

**Affiliations:** 1 Environnement and Health team, Polydisciplinary Faculty of Safi, Cadi Ayyad University, Safi, Morocco; 2 Laboratory of Anatomical Pathology, Faculty of Medicine and Pharmacy of Casablanca, Hassan II University, Casablanca, Morocco; 3 Cellular and Molecular Pathology Laboratory, Faculty of Medicine and Pharmacy of Casablanca, Hassan II University, Casablanca, Morocco; 4 Laboratory of Genetics and Cellular Enegineering, Faculty of Medicine and Pharmacy of Casablanca, Hassan II University, Casablanca, Morocco

**Keywords:** Anti-inflammation, *Capparis spinosa L*, Immunomodulation, CD4+ T cells, Th1, Th2 and Th17

## Abstract

**Background:**

Several chronic inflammatory diseases are characterized by inappropriate CD4+ T cell response. In the present study, we assessed the ability of *Capparis spinosa* L. (CS) preparation to orientate, in vivo, the immune response mediated by CD4+ T cells towards an anti-inflammatory response.

**Methods:**

The in vivo study was carried out by using the contact hypersensitivity (CHS) model in Swiss mice. Then we performed a histological analysis followed by molecular study by using real time RT-PCR. We also realized a phytochemical screening and a liquid-liquid separation of CS preparation.

**Results:**

Our study allowed us to detect a significantly reduced edema in mice treated with CS preparations relative to control. CS effect was dose dependent, statistically similar to that observed with indomethacin, independent of the plant genotype and of the period of treatment. Furthermore, our histology studies revealed that CS induced a significant decrease in immune cell infiltration, in vasodilatation and in dermis thickness in the inflammatory site. Interestingly, we showed that CS operated by inhibiting cytokine gene expression including IFNγ, IL-17 and IL-4. Besides, phytochemical screening of CS extract showed the presence of several chemical families such as saponins, flavonoids and alkaloids. One (hexane fraction) out of the three distinct prepared fractions, exhibited an anti-inflammatory effect similar to that of the raw preparation, and would likely contain the bioactive(s) molecule(s).

**Conclusions:**

Altogether, our data indicate that CS regulates inflammation induced in vivo in mice and thus could be a source of anti-inflammatory molecules, which could be used in some T lymphocyte-dependent inflammatory diseases.

**Electronic supplementary material:**

The online version of this article (doi:10.1186/s12906-017-1569-7) contains supplementary material, which is available to authorized users.

## Background

Many chronic inflammatory diseases are characterized by inappropriate or dysregulated CD4+ T cell response [1]. CD4+ T cells play a major role in the induction and regulation of immune responses, mainly by secreting cytokines. Given their central role in regulating innate and adaptive immunity, CD4+ T cells represent a key for both immune protection and immune pathology [1]. The discovery of a new CD4+ T cell subset, Th17, has transformed our understanding of the development of an increasing number of chronic immune-mediated diseases. Contact hypersensitivity (CHS) can be induced in animals; on which could be used as a model in which several basic immunological mechanisms can be studied [2]. Traditionally, CHS represents the prototype of delayed-type hypersensitivity, which is mediated by T cells [3–5]. In mice, CHS has been studied using haptens such as dinitrofluorobenzene (DNFB), FITC, and oxazolone [2, 6]. The CHS reaction consists of two distinct phases, the afferent phase and the efferent phase [5–7]. During the afferent or the sensitization phase, animals are epicutaneously exposed to haptens, the first contact of a hapten with skin leads to its binding to an endogenous protein in the skin where they form immunogenic hapten-carrier complexes [2]. The haptens induce local inflammation by acting on keratinocytes and innate immunity receptors. Activation of the skin innate immunity including keratinocytes induces the production of mediators (IL-18, IL-1β, TNF-γ, ATP, PGE2, LTB4, ROS, histamine, CCL20) by resident skin cells. These mediators are able to induce the recruitment, migration and activation of cutaneous antigen presenting cells (APC) [2, 6, 8]. The hapten-carrier complex is taken up by Langerhans cells (LCs) and dermal dendritic cells (dDCs) which migrate from the epidermis to the draining lymph node (in a CCR7-CCL19/CCL21-dependent manner), where they present the haptenated peptides to naive T cells which are subsequently activated [9, 10]. The newly activated T cells proliferate, resulting in the generation of effector/memory T cells and migrate out of the lymph node into circulation. During the efferent or the elicitation phase, animals are reexposed to the same hapten at a remote skin site. Once more, haptenated peptides are uptaken by skin APC, which present to hapten specific primed T cells patrolling in the skin. This results in the recruitment of antigen-specific T cells to the site of challenge and leads to T cell-mediated tissue damage [2, 6, 8]. This reaction involves both Th1 cells, which release pro-inflammatory cytokines, such as IFN-γ [3] and Th17 cells, which release IL-17 family cytokines [11, 12]. However, studies have revealed that the Th2 cells are necessary during the elicitation phase of the CHS reaction [5]. Therefore, many research efforts are focusing on the identification of anti-inflammatory agents able to effectively reduce the inflammatory mediators produced by CD4+ T cells.

The medicinal plants represent a rich source of biologically active compounds with potential therapeutic applications [13]. *Capparis spinosa* L*.* (CS) is a small shrub belonging to the family of the *Capparidaceae*, and the genus *Capparis*. It is grown in the Mediterranean region and also in the dry regions in West and central Asia [14]. In Morocco, CS is found abundantly in different regions especially in the regions of Fez, Taounate, Meknes, Marrakech and Safi [15, 16]. It has been suggested that CS would be a good candidate for new drug discovery [17]. Biological studies of various parts of this plant have revealed diverse bioactivities, including antihepatotoxic [18], anti-allergic and anti-histaminic [19], anti-oxidative [20, 21], anti-arthritic [22], hypolipidemic [23], and chondroprotective effects [24]. However, the immunomodulatory effect of CS is still not entirely established. Recently, we have shown that. non toxic doses of CS preparation induce an overall anti-inflammatory response in vitro in human PBMCs from healthy donors through significant inhibition of the proinflammatory cytokine IL-17 and induction of IL-4 gene expression [25]. In this study, we have used CS preparations to check whether they contain natural substances able to orient the immune response mediated by CD4+ T cells in vivo and thus generating an anti-inflammatory state. We found that CS inhibited the DNFB-mediated CHS reaction in mice. The anti-inflammatory effect observed with CS was independent of the plant genotype and of the period of treatment. Furthermore, the treatment with CS 24 h after the initiation of the disease also significantly suppressed the inflammation. It is noteworthy that CS anti-inflammatory effect was similar to that observed with the well-established anti-inflammatory compound, indomethacin. Histological studies showed an inhibition of cell infiltration to the inflammation site following treatment with CS. Interestingly, Real time PCR analysis revealed a suppression of cytokine gene expression, including the pro-inflammatory cytokine IL-17, in the draining lymph nodes of CS-treated mice. Finally, phytochemical analysis of CS preparation showed the presence of five chemical compounds; and it is suggested that the potential bioactive molecule is likely conserved in the hexane fraction.

## Methods

### Materials and reagents

2,4-dinitro-1-fluorobenzene (DNFB), acetone, potassium phosphate dibasic puriss (K2HPO4), potassium phosphate monobasic puriss (KH2PO4), dimethyl sulfoxide (DMSO) and potassium chlorid (Kcl) were obtained from SigmaAldrich. TRIzol reagent, SuperScript™ III Reverse Transcriptase, oligo(dT)12–18, RNaseOUT™ Recombinant RNase Inhibitor and the fluorescent SYBR Green Supermix from Invitrogen. Methanol, absolute ethanol, Acetic acid, Hexane and ethylacetate were from VWR PROLABO chemicals (BDH). Hematoxylin from Solvachim, Eosin gelblich from MERCK. Indomethacin was obtained from PHARMA 5. Digital caliper (Nobel), digital biological microscope (Motic), NanoVueTM Plus Spectrophotometer (GE Healthcare, UK) and Real-Time PCR system (Applied Biosystem FAST 7500) were used.

### Animals

Swiss albino mice (25-27 g) were used. They were obtained from the institute Pasteur of Casablanca-Morocco. Before initiation of experiments, the mice were acclimatized for a period of 7 days under standard environmental conditions. They have had free access to food and water and were kept in a room with 12 h day/night cycle. All efforts were made to minimize animals suffering and to reduce the number of animals used in the study. The project was approved by the Ethic committee for biomedical research of the Faculty of Medicine and Pharmacy of Casablanca, Hassan II University, Casablanca, Morocco. Under reference number, 07/16

### Contact hypersensitivity model

Unanesthetized Swiss mice were sensitized on Days 0 and 1 by applying 50 μl of 0.5% 2.4-dinitro-1-fluorobenzene (DNFB) dissolved in acetone/olive oil (4:1, v/v) on the shaved abdominal skin (positive control), negative control mice were shaved and painted with the acetone-olive oil mixture alone. Six days later, the baseline right ear thickness was measured with a digital caliper then the interior and external surfaces of right ears were challenged with 20 μl of 0.2% DNFB. Ear swelling was calculated as ear thickness 24 h after challenge (then every other 24 h as shown in the corresponding figures). Baseline ear thickness was subtracted from the obtained value. Ear thickness was measured in a blinded manner; all groups comprised five or seven animals.

### Plant material

The leaves of three specimen of *Capparis spinosa* L*.* were collected in August, from three stations in the surroundings of Safi region (in Morocco). The plant material was identified and a voucher specimen has been deposited under number 93664, in the Herbarium Chérifien Scientific Institute of Rabat, Morocco. The plant material was dried at room temperature.

### Extraction

The leaves were washed and dried under shade and manually crushed into powder. The powder was extracted by cold maceration method at room temperature using methanol or ethanol for 48 h to obtain the methanol or ethanol extract. The solvent extract was filtered using a millipore filter to remove particulate matter. The filtrate obtained was concentrated in rotary evaporator at 37 °C. This resulting preparation was used for the anti-inflammatory and phytochemical studies. The extract was conserved at 4 °C in the dark.

### *Capparis spinosa* L phenotyping

Morphological analysis was performed on the aerial parts of the sampled caper. Quantitative and qualitative traits were measured in leaves, flower buds and mature flowers, thorns and twigs stipular. For each sample, five replicates were measured and recorded, and the average was used in the subsequent analysis.

### *Capparis spinosa L* genotyping

Total DNA was extracted from the leaves of fresh and dried caper sampled in the three aforementioned stations according to the non-commercial basic protocol described by Doyle based on cationic detergent CTAB (Hexadecyltrimethyl ammonium bromide) modified [26, 27]. PCR reactions were performed using four primers:

- IMA12: 5’-CACACACACACACACATG-3’
- IMA303: 5’-(AGT)(AGC)(AGT)CA(CCA)4C-3’
- IMA834: 5’AGAGAGAGAGAGAGAGCTT-3’
- UBC818: 5’-CACACACACACACACAG-3’

Amplification reactions were performed in a thermal cycler TC-3000. The amplification conditions were as follows: initial denaturation step of 5 min (94 °C), 35 cycles of 30 s at 94 °C, 1 min at 52 to 66 °C (depending on the primer pair used), 1 min at 72 °C. The reaction was completed by a final elongation step of 7 min at 72 °C.

### Phytochemical analysis

The methanol extract was subjected to phytochemical analysis for constituent identification using the phytochemical methods, which were previously described [28]. In general, tests for the presence or absence of phytochemical compounds involved the addition of an appropriate chemical agent to the preparation in a test tube. The mixture is then vortexed. The presence or absence of saponins, flavonoids, tannins, alkaloids is subsequently detected.

### Fractionation

The methanol extract was subjected to fractionation with hexane and ethyl acetate. 7 g of the methanol extract was suspended in 20 ml distilled water at 35 °C and successively extracted with 40 ml of hexane for 10 min (×5) and 40 ml of ethyl acetate for 10 min (×4) by liquid-liquid extraction. At the end of the extraction, the three fractions, hexane (F1), ethyl acetate (F2) and aqueous fraction (F3) have been concentrated in a rotary evaporator respectively at temperatures of 35 °C, 35 °C and 40 °C. All the fractions (except F2, were solubilized with 5% DMSO) were solubilized with Phosphate-Buffered Saline (PBS) and tested for anti-inflammatory activity.

### Treatment protocol

The plant extract and fractions were solubilized in PBS and administered by intraperitoneal injection (i.p.) for 7, 4, 3 or 2 days at doses of 1.07 g/Kg and 0.428 g/Kg body weight for methanol extract; 1.07 g/Kg for ethyl acetate fraction and ethanol extract; 0.30 g/Kg for hexane fraction and 0.38 g/Kg for aqueous fraction. Another group received i.p. injections of indomethacin at a dose of 2 mg/kg for three consecutive days after challenge. Though indomethacin is sparingly soluble in PBS, a homogenous solution was achieved by constant agitation stirring. The volume used was of 100 μl. The control animal group received the same volume of PBS. Mice were randomly divided into eight groups (*n* = 5) as follows:

Group I: negative control (control -), mice were sensitized by mixture vehicle alone, challenged with DNFB and received i.p. injections of normal saline; Group II: positive control (control +), mice were sensitized and challenged with DNFB and received i.p. injections of normal saline; Group III: indomethacin (INDO), mice were sensitized and challenged with DNFB and received i.p. injections of indomethacin 24, 48 and 72 h after challenge; Group IV: methanol extract (CS Met or CS), mice were sensitized and challenged with DNFB and received i.p. injections of methanol extract on days -1, 0, 1, 2, 5, 6 and 7, surrounding sensitization and challenge; Group V: ethanol extract (CS Eth), mice were sensitized and challenged with DNFB and received i.p. injections of methanol extract on days -1, 0, 1, 2, 5, 6 and 7, surrounding sensitization and challenge; Group VI: methanol extract (CS.S), mice were sensitized and challenged with DNFB and received i.p. injections of methanol extract on days -1, 0, 1 and 2, surrounding sensitization; Group VII: methanol extract (CS.C), mice were sensitized and challenged with DNFB and received i.p. injections of methanol extract on days 5, 6 and 7, surrounding challenge; Group VIII: methanol extract (CS.T) or fractions, mice were sensitized and challenged with DNFB and received i.p. injections of extract 2 or 3 days after challenge on days 7, 8 and 9. Ear swelling was calculated as ear thickness after challenge minus ear thickness before challenge.

### Histology

48 h after challenge, immediately after sacrifice by cervical dislocation, the individual ears were collected by dissection and fixed in 10% phosphate buffered formalin for 48 h. The ears were thereafter dehydrated in graded concentrations of alcohol (70%, 80%, 90% and 100% x 2), cleared in toluene and embedded in paraffin at 60 °C. The paraffin-embedded tissue sections were cut on a microtome at 3 μm through the midsagittal plane, mounted on clean glass slides and dried for 30 min at 60 °C. The sections were stained with haematoxylin and eosin (H&E), and examined by Motic digital microscope. A certified pathologist analyzed the samples in a blinded manner. A minimum of three sections per animal experimentation was examined for the presence and degree of thickenes and inflammation of the epidermis and dermis. Digital photographs were taken at different magnification.

### Quantitative real time PCR

Total RNA was extracted using TRIzol reagent (Invitrogen). Twenty for hours after challenge, immediately after sacrifice, individual lymph nodes draining the inflammatory site were collected, frozen in the presence of Trizol at −80 °C, untiluse. Total RNA was extracted from the frozen tissue samples as described by the manufacturer. RNA concentration and quality were measured using the NanoVueTM Plus Spectrophotometer (GE Healthcare, UK). Then, total RNA was transformed to first strand complementary DNA (cDNA) by incubating with SuperScript™ III Reverse Transcriptase using oligo (dT)12–18 as primer. PCR was carried out with the gene-specific primers:

- INFγ sense, 5’-TGCATCTTGGCTTTGCAGCTCTTC-3’;
- INFγ antisense, 5’-GGGTTGTTGACCTCAAACTTGGCA-3’;
- IL-4 sense, 5’-AACACCACAGAGAGTGAGCTCGTCT-3’;
- IL-4 antisense, 5’-TGGACTCATTCATGGTGCAGCTTAT-3’;
- IL-17 sense, 5’-ATGCTGTTGCTGCTGCTGAGCC-3’;
- IL-17 antisense, 5’-GGTCTTCATTGCGGTGGAGAG-3’;
- *β*-actin sense, 5’-TGGAATCCTGTGGCATCCATGAAAC-3’;
- *β*-actin antisense, 5’-TAAAACGCAGCTCAGTAACAGTCCG-3’.

*β*-actin was used as an internal standard to evaluate relative expression of INFγ, IL-4 and IL-17. Expression level of each gene was measured in duplicate, in the presence of the fluorescent dye (iQ SYBR Green Supermix) using a Real-Time PCR system (Applied Biosystem FAST 7500). Experiments were performed in a 20 μL reaction volume with specific primer pairs, and the conditions of real-time quantitative PCR were as follows: denaturation at 95 °C for 15 s and amplification by cycling 40 times at 95 °C for 15 s, 60 °C for 30 s and 72 °C for 30 s. The values were represented as normalized expression: 2^−ΔCt^ (ΔCt = Ct target RNA – Ct β-actin).

### Statistical analysis

All the in vivo experiments consisted of five or seven mice, and all the other determinations were conducted in duplicate. The statistical significance between mean values was determined by using student’s *t*-test. One-way analysis of variance was used to test the difference between groups using SPSS software version 15.0.1 (Chicago, IL). P value˂0.05 was considered to be significant [* *p* < 0.05; ** *p* < 0.01; *** *p* < 0.001]. Data were expressed as a mean ± SD. Analysis of the identity between the three samples of caper was performed using similarity coefficients and dendrograms via PAST software version 1.74 (http://folk.uio.no/ohammer/past/).

## Results

### CS inhibited the DNFB-mediated CHS reaction

**Fig. 1:**
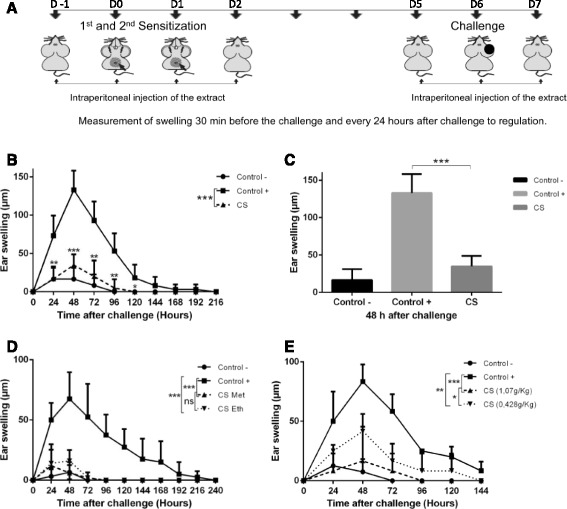
CS methanol extract decreased the CHS reaction. Mice were sensitized on the shaved ventral abdomen on days 0 and 1 by applying 50 μl of 0.5% DNFB (positive control ■), or were treated with the vehicle alone (negative control●); all groups of mice were challenged with 20 μl of 0.2% DNFB on the right ears on day 6. **a** Scheme for the experimental protocol. **b** Another group of mice was sensitized and challenged with DNFB and received i.p. injections of CS extract (▲) at a dose of 1.07 g/Kg. **c** Histograms representing ear swelling 48 h after challenge. **d** Mice received i.p. injections of either methanol (▲) or ethanol (▼) CS extracts at a dose of 1.07 g/Kg. **e** Mice received i.p. injections of CS methanol extract at doses of either of 1.07 g/Kg(▲) or 0.428 g/Kg body weight (▼) on days -1, 0, 1, 2, 5, 6 and 7. Data were expressed as averages of the values of ear swelling after the challenge. *P* value <0.05 was considered to be significant [* *p* < 0.05; ** *p* < 0.01; *** *p* < 0.001]. Data are representative of 3 or 2 number of experiments with *n* = 5 of mice per group (Except the negative control) (Additional file 1)

To evaluate the anti-inflammatory effect of CS extract in vivo, female «Swiss» mice were used. Mice were sensitized and challenged with DNFB and received i.p. injections of either Phosphate-Buffered Saline (PBS) (positive control) or of CS extract dissolved in PBS at a predetermined optimal dose of 1,07 g/Kg on days -1, 0, 1, 2, 5, 6 and 7, encompassing both sensitization and challenge steps as depicted in (Fig. 1a). Negative control mice received i.p. injections of PBS or were left untreated. The effect of CS extract on CHS progression was compared with the positive control group. After challenge, ear thickness was measured as a marker for clinical manifestation of CHS severity. Treatment with CS was able to regulate the CHS response in mice significantly (*P* < 0.001) at the level of ear in comparison with control, with an inhibition percentage of approximately 73.44% (Fig. 1b, c). To check if the protective effect of CS was influenced by the extraction solvent, or by the doses of the used extract, mice were sensitized and challenged with DNFB and received i.p. injections of methanol extract at doses of 1.07 g/Kg and 0.428 g/Kg body weight, or ethanol extract at doses of 1.07 g/Kg. Anti-inflammatory effect of ethanol CS extract has been observed with differences, which were statistically significant, compared with the positive control group. Swelling of the right ear in the positive control group persisted 10 days after the challenge, while the swelling was resolved after 4 days of the challenge for CS-treated groups. However, the difference observed between groups treated with the methanol and ethanol CS extracts was not statistically significant (Fig. 1d). However, the difference observed between the group treated with the methanol extract at doses of 1.07 g/Kg and at doses 0.428 g/Kg was statistically significant (Fig. 1e). The peak of swelling of the right ear (challenged) was 83 μm in the positive control group, while those in the groups treated with methanol extracts, at doses of 0.428 g/Kg and 1.07 g/Kg body weight, did not exceed 41 and 16 μm respectively. The results suggest that CS significantly inhibited edema in mice and exhibits anti-inflammatory activities in a dose-dependent manner. Then we wondered whether the observed anti-inflammatory effect of CS depends on the plant variety.

### The anti-inflammatory effect of CS was independent of the plant genotype and of the period of treatment

**Fig. 2:**
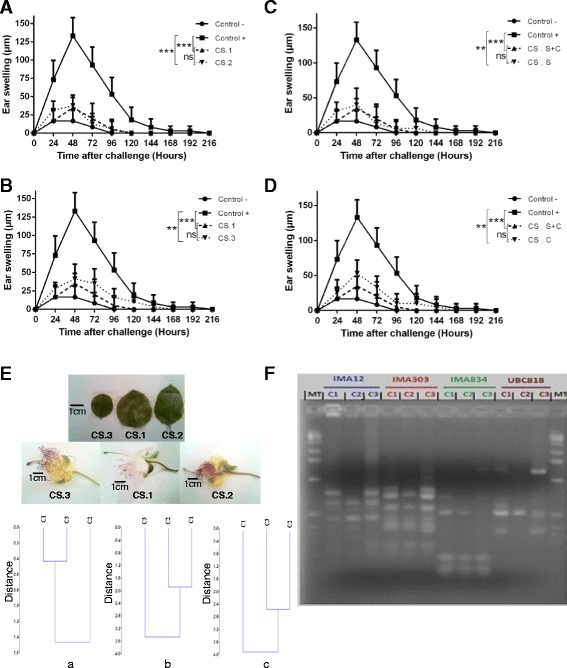
The anti-inflammatory effect of CS is independent of the plant genotype and of the period of treatment. Different Groups of mice (≈5 mice per group) were sensitized on the shaved ventral abdomen on days 0 and 1 by applying 50 μl of 0.5% DNFB (positive control ■), or with the vehicle alone (negative control●) and were all challenged with 20 μl of 0.2% DNFB on the right ears on day 6. Other groups were sensitized and challenged with DNFB and received i.p. injections of the first (CS. 1), second (CS.2) or third (CS.3) CS genotypes (**a** and **b**). In another series of experiments (**c** and **d**), mice received i.p. injections of CS extract on days -1, 0, 1 and 2 during the period of sensitization (s) or on days 5, 6 and 7, during the period of the challenge (c) at a dose of 1.07 g/Kg body weight or during the periods of sensitization and challenge (s + c). Data were represented as averages of ear swelling values after the challenge. *P* value <0.05 was considered to be significant [* *p* < 0.05; ** *p* < 0.01; *** *p* < 0.001]. **e** Genetic comparison of the three varieties of CS presenting with morphological differences. (a): quantitative descriptors, (b): qualitative descriptors and c: Qualitative versus quantitative descriptors and Photographs of leaves and flowers of these plant varieties. **f** Molecular profil of samples based on 4 distinct ISSR primers (Additional file 2)

Based on Morphological and molecular characterization we showed that the three specimens of caper studied correspond to three different genotypes (Fig. 2e, f). In order to determine whether the anti-inflammatory effect of CS preparation could be maintained indifferent plant genotypes, presenting with phenotypic and genotypic differences (Fig. 2e, f), we compared the effect of our primary extract (genotype 1) with two other genotypes (2 and 3). Groups of mice sensitized and challenged with DNFB using the previously described protocol, were treated with sample 1, sample 2 or sample 3 of methanol extracts on days -1, 0, 1, 2, 5, 6 and 7. Measures of edema, after the challenge, suggested that the three samples of CS show a protective effect. The three samples significantly suppressed the CHS response, but with differences not statistically significant (Fig. 2a, b). This finding suggests that the anti-inflammatory effect of CS extract is not influenced by the phenotypic and genotypic differences of the plant.

We have previously shown that treatment with CS extract, encompassing sensitization and challenge; inhibited DNFB-mediated CHS reaction. The pathophysiology of classical CHS is well-known and requires two temporally dissociated steps: the afferent phase (sensitization) and the efferent phase (challenge or elicitation). In order to get insight into the mechanism of anti-inflammatory effect observed of CS extract, we analyzed the anti-inflammatory effect of CS extract in each phase. Experiments were carried out according to the protocol described in materials and methods. The results suggest that the inhibition observed with treatments at the level of the two phases of the induction of the disease (73.57%) is similar to that observed after treatment during the sensitization phase only (69,63%) (Fig. 2c). The inhibition observed with the treatment at the time of the challenge only, is less important (60.15%), but also not statistically significant (Fig. 2d). In conclusion, CS preparation presents similar inhibition of the CHS reaction in the three distinct situations (administration of the extract at the time of the sensitization only or at the time of the challenge only or at the level of the two phases). The question arose whether CS would also be effective in inhibiting an already induced CHS response.

### Treatment with CS 24 h after the initiation of CHS reaction also significantly suppressed the inflammation

**Fig. 3:**
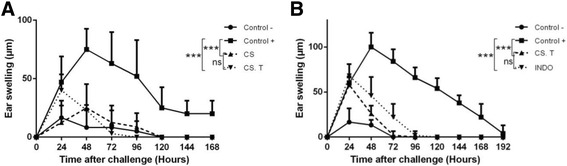
Treatment with CS extract following the induction of CHS reaction also significantly inhibited the inflammation. Different Groups of mice (≈5 mice per group) were sensitized on the shaved ventral abdomen on days 0 and 1 by applying 50 μl of 0.5% DNFB (positive control ■), or with the vehicule alone (negative control ●) and were all challenged with 20 μl of 0.2% DNFB on the right ears on day 6. Other groups were sensitized and challenged with DNFB and received i.p. injections of CS extract on days -1, 0, 1, 2, 5, 6 and 7(panel **a**, CS); or on days 7, 8 and 9, following the induction of CHS reaction (panel A, CS.T) (▼). In other experiments (panel **b**), mice received i.p. injections of CS extract (▲) or Indomethacin (▼) at a dose of 1.07 g/Kg and 2 mg/Kg respectively for three consecutive days following the challenge (24 h, 48 h and 72 h). Data were represented as averages of ear swelling values after the challenge. *P* value <0.05 was considered to be significant [* *p* < 0.05; ** *p* < 0.01; *** *p* < 0.001] (Additional file 3)

Mice were sensitized with DNFB on the abdomen in the absence of CS and were ear challenged 6 days later, resulting in an efficient ear swelling response. On days 7, 8 and 9, third of mice received i.p. injection of CS, another third received i.p. injection of the anti-inflammatory drug Indomethacin and another third received i.p. injection of PBS for positive control. Another group of mice received i.p. injection of CS during both sensitization and challenge phases (Fig. 3). The treatment with the anti-inflammatory drug Indomethacin and CS, reduced significantly the ear swelling in comparison with the vehicle-treated positive control group (*P* < 0.001). However, the difference observed between the group treated with CS (24 h after disease induction) and the group treated with CS during sensitization and challenge and the group treated with Indomethacin is not statistically significant (Fig. 3a, b). In conclusion, it is suggested that CS effect is similar to that of a well-established anti-inflammatory, Indomethacin. On the other hand, the anti-inflammatory effect of CS persists even when applied after disease induction.

### CS regulated inflammation induced in vivo in mice through inhibition of cell infiltration and cytokine gene expression

In order to better understand the mechanisms underlying the anti-inflammatory effects observed, we assessed the effect of CS extract on the infiltration of immune cells in the inflammation site, 48 h after the challenge.

**Fig. 4:**
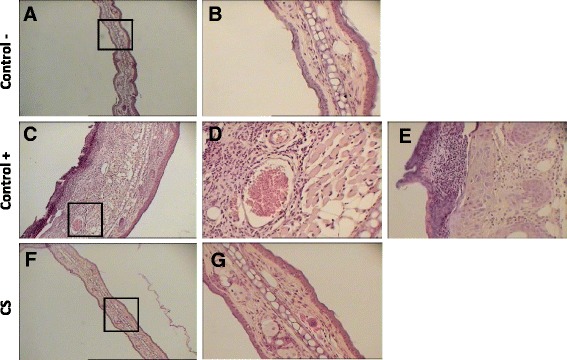
Histological analysis of the skin from the inflammatory site. Paraffin-embedded sections of inflamed skin from negative control (**a** and **b**), positive control (**c**, **d** and **e**) and CS-treated (**f** and **g**) mice at 100× and 400×. Mice were sacrificed 48 h after the challenge, and stained with H&E after formalin fixation and paraffin inclusion (Additional file 4)

**Fig. 5:**
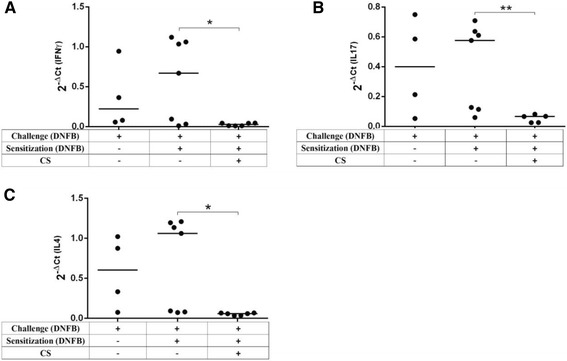
CS inhibited cytokine gene expression in the lymph nodes draining the inflammatory site. Expression of mRNA was investigated by real-time PCR in positive control mice (sensitized and challenged with DNFB “0.5 and 0.2%”, *n* = 7), in negative control mice (only challenged by 0.2% of DNFB, *n* = 4), and in mice sensitized and challenged with DNFB “0.5 and 0.2%” and treated with CS at this two phases (Cap, *n* = 6). The analysis of IFNγ (**a**), IL-17 (**b**) and IL-4 (**c**) mRNA expression is shown. Results are represented as normalized expression: 2 − ΔCt (ΔCt = Ct RNA target – Ct β-actin). *P* value <0.05 was considered to be significant [* *p* < 0.05; ** *p* < 0.01; *** *p* < 0.001] (Additional file 5)

Histopathological analysis of the positive control group showed anintensified inflammation manifested by increase of ear thickness (edema), excessive inflammatory cell infiltration, swelling of fibroblasts, dilatation of papillary vessels (vasodilatation), with perivascular lymphohistiocytic infiltrate, thickening of the dermis and epidermal hypertrophy (Fig. 4c, d, e). These features are recognized as the microscopic hallmark of contact dermatitis [28]. Interestingly, our study demonstrated that this DNFB-induced features of inflammation were also suppressed upon CS treatment, as shown in Fig. 4f, g. CS induced a significant decrease in immune cell infiltration, epidermal hypertrophy, dermis thickness, swelling of fibroblasts and vasodilatation. Indeed, the anti-inflammatory effect of CS on CHS response was further corroborated by histological examination of the inflamed ear tissue. As T cells are known to be the critical cell type mediating the CHS response, we then investigated whether T cell function was affected. In order to assess the immuno-modulatory efficacy of CS extract used in this study, the relative expression of the three key cytokines IFNγ (for Th1 cells), IL-4 (for Th2 cells) and IL-17 (for Th17 cells) in CHS reaction induced by DNFB, was investigated by RT-qPCR. CHS and expression of IFNγ, IL-17 and IL-4 mRNA was markedly and significantly suppressed in mice treated with CS relative to the control (Fig. 5). These findings suggest that CS likely operated by inhibiting cytokine gene expression. A chemical analysis was subsequently performed in order to identify the fraction of the extract, which contains the bioactive compound(s).

### Phytochemical screening and identification of the potential anti-inflammatory fraction of the CS extract

**Fig. 6:**
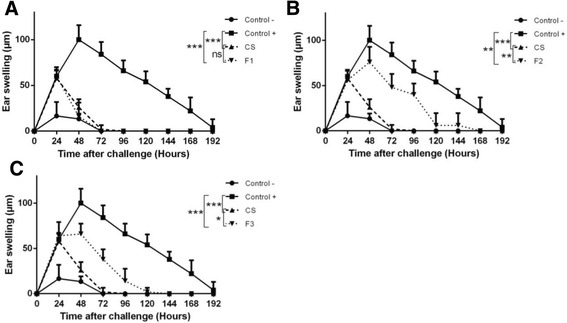
The hexane fraction of CS reproduced the anti-inflammatory effect. Mice (5 mice per group) were sensitized on the shaved ventral abdomen on days 0 and 1 by applying 50 μl of 0.5% DNFB (positive control ■), or were treated with the vehicle alone (negative control●); all groups of mice were challenged with 20 μl of 0.2% DNFB on the right ears on day 6. Other groups of mice were sensitized and challenged with DNFB and received i.p. injections of crude extract of CS (▲) (panel **a**, CS), or the hexane fraction of CS (panel **a**, F1), the ethyl acetate fraction (panel **b**, F2) or the aqueous fraction (panel **c**, F3) (▼). Data were represented as averages of ear swelling values after the challenge. *P* value <0.05 was considered to be significant [* *p* < 0.05; ** *p* < 0.01; *** *p* < 0.001] (Additional file 6)

The preliminary extraction of CS leaves with aqueous methanol extract, which on phytochemical analysis, showed the presence of five known compounds were identified as: alkaloids, flavonoids, phenolic compound, saponins and antraquinones; as shown in Table 1. The crude extract was subjected to fractionation with hexane and ethyl acetate, by liquid-liquid extraction. Extraction of the plant material using different solvents gave three fractions, hexane fraction (F1), ethyl acetate fraction (F2) and aqueous fraction (F3). All fractions showed significant anti-inflammatory activity, with varying degrees of anti-inflammatory activities (Fig. 6). Only one fraction showed very high activity. This hexane fraction (F1) exhibited anti-inflammatory effect similar to that of the raw extract, with differences not statistically significant (Fig. 6a). In conclusion, it is suggested that the potential bioactive molecule of CS extract is probably conserved in hexane fraction.

**Table 1:** Phytochemical screening of the methanol extract of aerial parts of *Capparis spinosa* L

Class of compounds	Alkaloids	Tannins	Flavonoids	Phenolic compound	Coumarins	Saponins	Anthocyanin	Antraquinones
Methanol extract	**+**	***-***	***+***	***+***	***-***	***+***	***-***	***+***

+ : Presence of constituents; – : Absence of constituents

## Discussion

In the present study, we examined the anti-inflammatory effect of CS extract in vivo using the animal model of contact hypersensitivity which represents a delayed-type hypersensitivity reaction, which is mediated by hapten-specific T cells [29]. Our results showed a significant anti-inflammatory action of CS. The effect was dose dependent and was not influenced by the treatment period or by the genotype of the plant species. This effect was observed even when treatment was applied after disease induction and it was similar to that of indomethacin, used as a positive control. Histology studies also revealed that CS induced a significant decrease in immune cell infiltration, in dermis thickness and in vasodilatation. Using RT-qPCR, we showed that this extract likely operated by inhibiting cytokine gene expression including IL-17, IFNγand IL-4. Chemical analysis showed the presence of saponins, flavonoids, alkaloids, phenolics and anthraquinones in the studied extract. Furthermore, fractionation of the extract suggested that the potential bioactive(s) molecule(s) is (are) likely conserved in the hexane fraction.

The in vivo assays allowed us to reveal a significant anti-inflammatory effect of methanol and ethanol leaf CS extracts, with an inhibition percentage of approximately 73.44%. This protection effect was dose dependent (Fig. 1). The aerial parts and fruit aqueous extracts of CS, were also found to exhibit significant anti-inflammatory activities against carrageenan-induced edema in rats [30, 31]. In other reports, it was also shown that aerial parts, fruits and flower buds of CS also exhibit immuno-modulatory properties [30–34]. These independent studies were using the same species but carried out in different places and thus probably using distinct varieties such as those described earlier, *Capparis spinosa L.var. intermis turra* and *Capparis spinosa L.var. aegyptia* [16, 35, 36]. To verify this hypothesis, we compared the effect of three different varieties of this plant, with phenotypic and genotypic differences; the initial primary variety (genotype 1) with two other related varieties (genotypes 2 and 3). Measures of edema, after the challenge, suggested that the three samples significantly inhibited the CHS response in mice, and with differences that are not statistically significant. This finding suggests that the anti-inflammatory effect of CS extract is not influenced by the phenotypic and genotypic variability of this plant (Fig. 2). In order to better understand the mechanisms underlying CS anti-inflammatory effect and taking into consideration the fact that LCs have been considered to be central in the initiation of CHS reaction in the sensitization phase [9, 37–39], we analyzed the effect of CS in each phase separately (sensitization and challenge). Our data indicated that CS exhibits similar degree of inhibition in the two situations (Fig. 2).

As antigen presentation is required for both sensitization and elicitation phases of CHS, and in both cases LC could be involved, we decided to evaluate the effect of CS later after disease induction. In these conditions, we showed that the anti-inflammatory effect of CS persisted (Fig. 3). This observation suggests that CS likely acts independently from LCs. This led us to think that CS could act on subsequent steps of the induction of CHS such as T cell activation and/or recruitment into the inflammatory site.

Upon induction of CHS in mice, the pinna of the ear is typically utilized to evaluate the inflammatory response, which peaks at 24-48 h after challenge, then progressively decreases through active down-regulating mechanisms [2, 5, 6]. Therefore, we assessed the effect of CS 48 h after the induction of CHS reaction. Histopathological analysis of the skin sections from positive control mice (treated with DNFB) showed epidermal hypertrophy (thickening of the epidermis), vasodilatation, swelling of fibroblasts and inflammatory cell infiltrate. These features were not observed in the skin from negative control mice and were all significantly decreased in the skin of the group of mice treated with CS (Fig. 4). A study was undertaken to evaluate the effect of CS leaves on the testicular tissue and epididymis in normal and trichloroacetic acid intoxication mice. This study revealed that leaf powder of CS and honey attenuated hyperplasia mononuclear cell infiltration and edema in the epididymis of mice [40]. This observation is in agreement with the present study and confirms that treatment of mice with CS extract suppresses inflammation, likely by inhibition of immune cell infiltration.

Since our data suggested that CS likely operated by inhibition of immune cell infiltration and not through LCs, we hypothesized that CS may act in on T cell mediators. Our study revealed that CS induced a significant decrease in the expression of pro-inflammatory cytokines IFNγ and IL-17 but also of the anti-inflammatory cytokine IL-4, compared with the positive control group of mice (mice sensitized and challenged with DNFB) (Fig. 5). In these experiments, the increased expression of cytokines detected in negative control groups might be due to the single treatment with DNFB (challenge) in this group of mice. This is the first study, at our knowledge, showing that CS regulates CHS reaction through inhibition of immune cell infiltration to the skin and suppression of cytokine gene expression. This improves the understanding of CS mechanism of action and provides new insights into therapeutic strategies for CHS or any pathology, which is mediated by proinflammatory cytokinen, IFNγ and IL-17. In agreement with the present study, we have recently shown that CS significantly inhibited the expression of IL-17 in vito in human PBMCs from healthy donors [25]. However, regulation of IL-4 by CS showed different results when this was applied either in vitro in human cells or in vivo in mice. While, CS showed an increase of IL-4 gene expression in vitro, it rather revealed a suppression of IL-4 gene expression in vivo in mice. Several points could account for this apparent discrepancy, including human versus mice cells, in vitro versus in vivo analysis. It has been reported, using MTT assay, that protein extracts of the fruit of CS exhibits a significant immunosuppressive activity through the inhibition of proliferation of splenocytes upon stimulation by Con-A [34]. It is likely that the observed inhibition of CS on cytokine expression is due to inhibition of proliferation of T cells. It has also been found that the in vitro exposure of human peripheral blood mononuclear cells (PBMCs) to a methanol extract of CS buds interferes with HSV-2 replication in PBMCs inhibiting the extracellular virus release by up-regulating the expression of IL-12, IFN-γ and TNF-α [32]. These results appear inconsistent with the previously cited. This could be due to an inter-species difference; since the former studies were performed in mice while the latter in human cells. The apparent discrepancy could also be due to a difference in the bioactive substances present in CS buds versus CS leaves. On the other hand, a protein exhibiting an N-terminal amino acid sequence purified from fresh CS seeds, inhibited proliferation of hepatoma HepG2 cells, colon cancer HT29 cells and breast cancer MCF-7 cells with an IC50 of about 1, 40 and 60 mM, respectively [41].

The extraction of CS leaves with methanol showed the presence of five compounds: alkaloids, flavonoids, phenolic compound, saponins and antraquinones. It has indeed been reported that biflavonoids from CS fruits inhibit NF-κB [33], a transcription factor known to play important roles in various biological processes including inflammation. NF-κB is also required for activated T cell survival, proliferation and cytokine gene expression [42]. Inhibition of NF-κB activity prevents GATA-3 expression and Th2 cytokine production (IL-4, IL-5 and IL-13) [43]. It also inhibits production of IFNγ by Th1 cells [42]. Another study supports the importance of NF-κB activation in Th17 cell differentiation [44]. Therefore, one possibility could be that CS inhibits cytokine expression by the inhibition of NF-κB activation. HPLC analysis performed by our team on CS preparation revealed the presence of polyphenol compounds including catechin, caffeic acid, syringic acid, rutin and frulinic acid [25].

In our experiments, when the crude extract was separated into three fractions, the hexane fraction has showed anti-inflammatory effect similar to that of raw extract. Then it is likely that the active molecule is whithin this fraction. Further experiments are underway in order to identify and isolate the bioactive molecule involved.

## Conclusion

The current study showed that CS preparation is a potential source of anti-inflammatory agents. CS inhibited inflammation by down-regulating the expression of pro-inflammatory cytokines and by inhibiting immune cell infiltration in the inflammation site. These findings could be a basis for its potential use in some T cell-mediated pathologies. Further investigations are, however, needed in order to identify and characterize the bioactive compound(s).

## Additional files

Additional file 1: Raw data relative to Fig. 1. (XLSX 14 kb)
Additional file 2: Raw data relative to Fig. 2 (XLSX 15 kb)
Additional file 3: Raw data relative to Fig. 3. (XLSX 11 kb)
Additional file 4: Raw data relative to Fig. 4. (DOCX 2993 kb)
Additional file 5: Raw data relative to Fig. 5. (XLSX 21 kb)
Additional file 6: Raw data relative to Fig. 6. (XLSX 13 kb)

## Abbreviations

ANOVA: Analysis of variance
APC: Antigen presenting cells
ATP: Adenosine triphosphate
CCL: Chemokine ligand
CCR: Chemokine receptor
CHS: Contact hypersensitivity
Con A: Concanavalin A
CS: *Capparis spinosa* L.
CS Eth: Ethanol extract of *Capparis Spinosa* L.
CS Met: Methanol extract of *Capparis Spinosa* L.
CTAB: Hexadecyltrimethyl ammonium bromide
CXCL: CXC chemokine ligand
dDCs: Dermal dendritic cells
DMSO: Dimethylsulfoxide
DNFB: 2,4-dinitro-1-fluorobenzene
H&E: Hematoxylin and eosin
HPLC: High performance liquid
HSV: Herpes simplex virus
i.p.: Intraperitoneal
IC50: 50% inhibition/inhibitory concentration
IFN: Interferon (e.g., IFN-γ)
IL: Interleukin (e.g., IL-4)
INDO: Indomethacin
LCs: Langerhans cells
LTB4: Leukotriene B4
mRNA: Messenger RNA
MTT: 3-(4,5-dimethylthiazol-2-yl)-2,5-dimethyltetrazolium bromide
NF-κB: Nuclear factor κB
*p*: Probability
PBMC: Peripheral blood mononuclear cell
PBS: Phosphate-buffered saline
PCR: Polymerase chain reaction
PGE2: Prostoglandin E2
RNA: Ribonucleic acid
ROS: Reactive oxygen species
RT-PCR: Reverse transcriptase polymerase chain reaction
RT-qPCR: Quantitative reverse transcription PCR
SD: Standard deviation
Th cell: T helper cell
TNF: Tumor necrosis factor
v/v: volume to volume
μl: Microliter (only with numbers)
