# Supplementary figures and images for: Anti-inflammatory potential of Capparis spinosa L. in vivo in mice through inhibition of cell infiltration and cytokine gene expression
Source: BMC Complement Altern Med. 2017 Jan 31;17:81. doi: 10.1186/s12906-017-1569-7 (PMC5282892; doi:10.1186/s12906-017-1569-7)

Fig.4

Control +


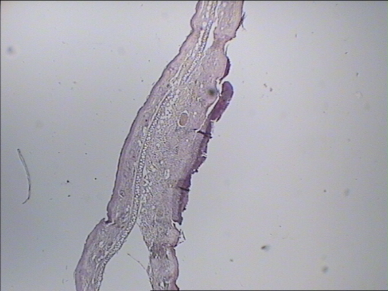

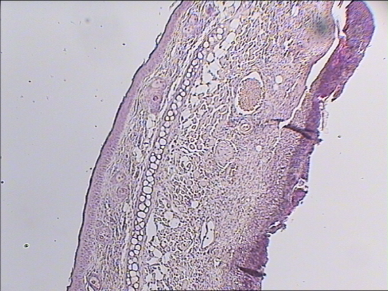

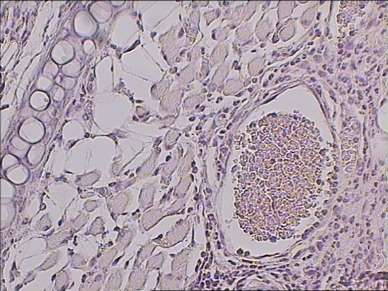


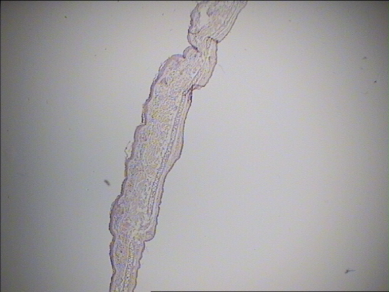

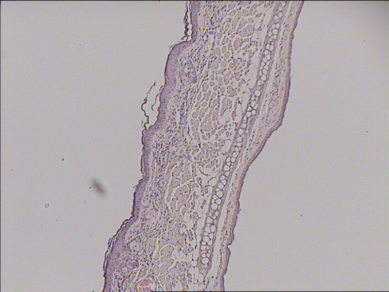

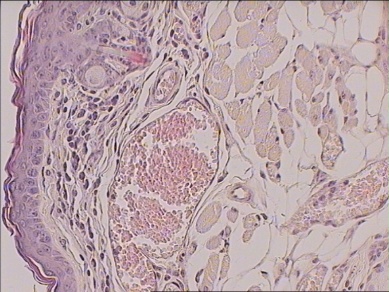


Control –


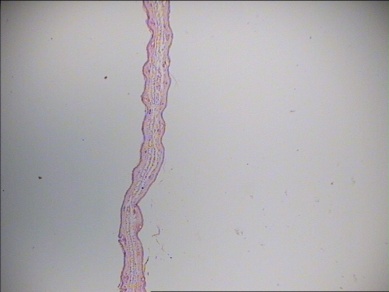

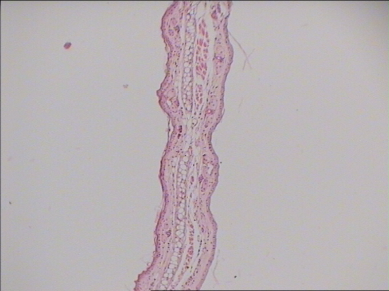

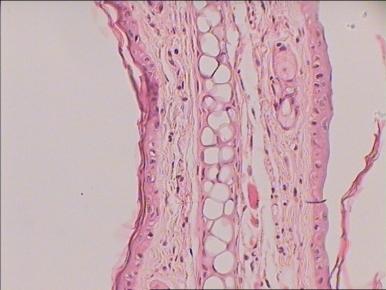


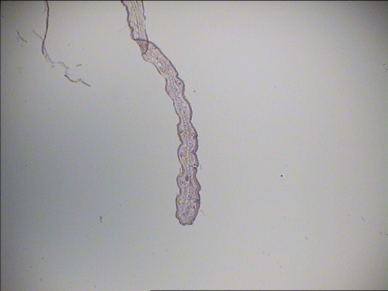

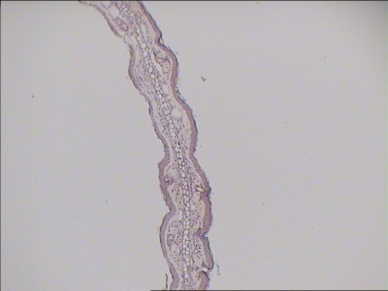

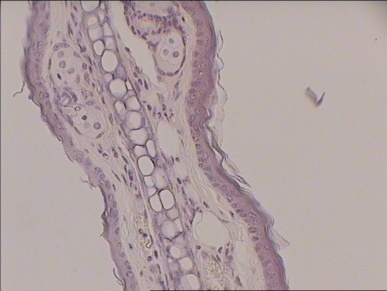


CS


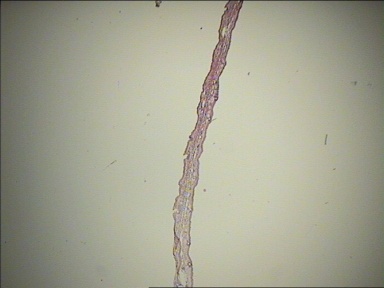

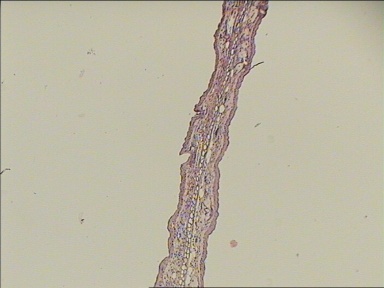

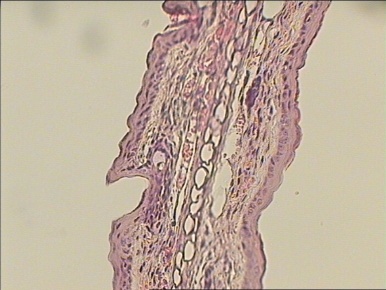


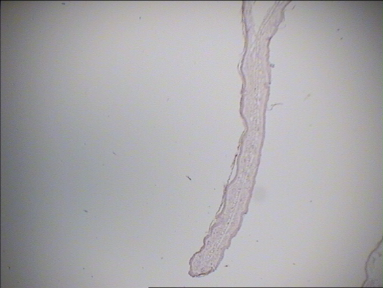

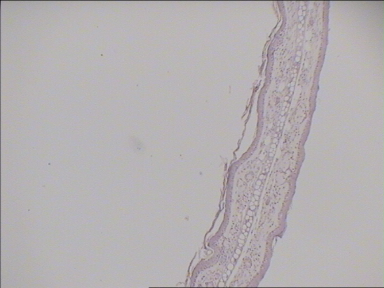

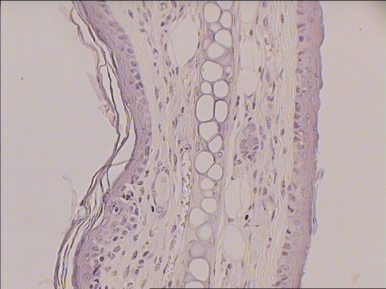

Supplement: Additional file 4: — Raw data relative to Fig. 4. (DOCX 2993 kb) [file 12906_2017_1569_MOESM4_ESM.docx]
